# Low Prevalence of Syndromic Respiratory Tract Infections among Returning Hajj Pilgrims Amidst the COVID-19 Pandemic: A Post-Hajj Survey

**DOI:** 10.3390/tropicalmed7080182

**Published:** 2022-08-13

**Authors:** Hashim A. Mahdi, Fadi S. Qashqari, Sumyya H. Hariri, Shahad Bamerdah, Shahad A. Altayyar, Hazim M. Almalki, Fayez A. Alwadani, Renan A. Alabbasi, Mohammed H. Alqahtani, Mohammad Alfelali, Ramon Z. Shaban, Robert Booy, Harunor Rashid

**Affiliations:** 1 Public Health Department, College of Health Sciences, Saudi Electronic University, Jeddah 23442, Saudi Arabia; 2 The Children’s Hospital at Westmead Clinical School, Faculty of Medicine and Health, The University of Sydney, Westmead, NSW 2145, Australia; 3 National Centre for Immunisation Research and Surveillance, The Children’s Hospital at Westmead, Westmead, NSW 2145, Australia; 4 Microbiology Department, Faculty of Medicine, Umm Al-Qura University, Makkah 24381, Saudi Arabia; 5 Faculty of Medicine, Umm Al-Qura University, Makkah 24381, Saudi Arabia; 6 Faculty of Medicine, Umm Al-Qura University, Qunfudhah 28821, Saudi Arabia; 7 Family and Community Medicine Department, Faculty of Medicine in Rabigh, King Abdulaziz University, Jeddah 25732, Saudi Arabia; 8 New South Wales Biocontainment Centre, Western Sydney Local Health District, Westmead, NSW 2145, Australia; 9 Susan Wakil School of Nursing and Midwifery, Faculty of Medicine and Health, The University of Sydney, Sydney, NSW 2050, Australia; 10 Centre for Population Health, Western Sydney Local Health District, North Parramatta, NSW 2151, Australia; 11 Sydney Institute for Infectious Diseases, The University of Sydney, Westmead, NSW 2145, Australia

**Keywords:** COVID-19, Hajj, influenza-like illnesses, pilgrims, respiratory tract infections

## Abstract

This study estimates the point prevalence of symptomatic respiratory tract infections (RTIs) among returned Hajj pilgrims and their contacts in 2021. Using the computer-assisted telephone interview (CATI) technique, domestic pilgrims were invited to participate in this cross-sectional survey two weeks after their home return from Hajj. Of 600 pilgrims approached, 79.3% agreed to participate and completed the survey. Syndromic definitions were used to clinically diagnose possible influenza-like illnesses (ILI) and COVID-19. Median with range was applied to summarise the continuous data, and frequencies and proportions were used to present the categorical variables. Simple logistic regression was carried out to assess the correlations of potential factors with the prevalence of RTIs. The majority of pilgrims (88.7%) reported receiving at least two doses of the COVID-19 vaccine before Hajj. Eleven (2.3%) pilgrims reported respiratory symptoms with the estimated prevalence of possible ILI being 0.2%, and of possible COVID-19 being 0.4%. Among those who were symptomatic, five (45.5%) reported that one or more of their close contacts had developed similar RTI symptoms after the pilgrims’ home return. The prevalence of RTIs among pilgrims who returned home after attending the Hajj 2021 was lower compared with those reported in the pre-pandemic studies; however, the risk of spread of infection among contacts following Hajj is still a concern.

## 1. Introduction

The Hajj pilgrimage is an annual religious mass gathering (MG) event in Makkah city, Kingdom of Saudi Arabia (KSA). During a pre-pandemic year, up to 3 million Muslims spent extended time at multiple sites in Makkah to perform various religious rituals [1]. This Hajj MG is associated with a high burden of infectious diseases, with respiratory tract infections (RTIs) being the most prevalent infections reported among the pilgrims [2,3]. The prevalence of influenza-like illnesses (ILI) among Hajj pilgrims ranges from 8% to 63% depending on the nationality of pilgrims, the study year and how ILI is defined [4,5].

Several respiratory viruses have emerged over the last several years such as Middle East respiratory syndrome coronavirus (MERS-CoV), which posed a great public health threat to the Hajj pilgrimage; nevertheless, no major outbreaks have been documented to date [6]. For instance, studies revealed no detection of MERS-CoV infection in any samples tested for viral RTIs obtained from 81 returning pilgrims from Bangladesh and 202 from the UK who presented with symptoms of respiratory illness [7,8]. Most recently, COVID-19 has been a major public health and infection control challenge at Hajj [9]. In order to attend the 2021 Hajj amidst the pandemic, definite eligibility criteria were set for residents of all nationalities inside KSA who were aged between 18 and 65 years, and only residents immunised against COVID-19 were permitted [10,11]. Furthermore, various non-pharmaceutical control measures were put in place during the pilgrims’ entire Hajj journey to limit the spread of SARS-CoV-2, including obligatory face mask use, advice on practising frequent hand hygiene, providing required hygienic products to pilgrims and maintaining physical distance [10,11].

Hajj and other MGs not only amplify the risk of infections among participants but also accelerate the transmission of these infections among their close contacts, making MGs a significant risk to global health security [6,12]. Only a limited number of studies assessed the prevalence of RTIs among pilgrims after their home return. Among returning UK pilgrims with ILI in 2005, 11% tested positive for a virus on RT-PCR, 9% for respiratory syncytial virus (RSV) type A and 2% for influenza A/H3 [13], while a 2009 survey among 1507 domestic pilgrims from Riyadh, KSA, showed that 97% of pilgrims reported RTI symptoms on their return from Hajj [14].

Data are scarce on the post-Hajj prevalence of RTIs, and to the best of our knowledge, no study has assessed the prevalence of symptomatic RTIs among pilgrims during Hajj amidst the COVID-19 pandemic. A study conducted by our team during the peak period of Hajj in 2021 demonstrates the need for a post-Hajj surveillance study [15]. To this end, this manuscript aims to report the point prevalence of syndromic RTIs among domestic Hajj pilgrims who returned home after the pilgrimage in 2021 and assesses the risk of RTIs among pilgrims’ contacts.

## 2. Materials and Methods

A cross-sectional study was conducted among returning domestic pilgrims (i.e., current residents of KSA) who attended Hajj (18–23 July) in the year 2021. Pilgrims of both genders aged 18 years or above were eligible to participate in the survey, while those who declined to participate or did not speak Arabic or English were excluded. Ethics approval was obtained from the Biomedical Ethics Committee of Umm Al-Qura University, Makkah, KSA (reference number: HAPO-02-K-012-2021-07-708), and all participants provided voluntary informed consent.

The survey was conducted using computer-assisted telephone interviews (CATI). A convenience sample of 600 pilgrims was contacted by phone from a list provided by Hajj hamlahs (companies that specialise in Hajj services and tourism) two weeks after their return from Hajj. Pilgrims who responded to the calls were interviewed using a structured questionnaire, and their responses were recorded in Microsoft Forms, an electronic survey development cloud-based software (Microsoft Office 356, version 2002, Redmond, WA, USA). The questionnaire consisted of two major parts: part one captured data about participants’ non-identifiable socio-demographics including the presence of pre-existing comorbidities, smoking status, and receipts of influenza and COVID-19 vaccinations, while part two focused on collecting information related to the occurrence of respiratory symptoms and some constitutional or gastrointestinal symptoms. In addition, pilgrims who developed symptoms were asked about the onset date and duration of these symptoms, their history of contact with room-/tent-mates suffering from RTIs during Hajj and whether any of their close contacts at home or work who were not pilgrims developed similar symptoms following any pilgrims’ home return.

A case of RTI was clinically defined as any pilgrim who reported having at least one respiratory symptom, including cough, sore throat, rhinitis, dyspnoea or smell or taste dysfunction at any time between the conclusion of Hajj rituals and the day of recruitment (i.e., within two weeks of pilgrims’ return to their home cities). The syndromic definitions for possible ILI and possible COVID-19 were applied according to criteria described and assessed elsewhere [16,17]. We used the ILI definition described by Rashid et al. as ‘a triad of subjective fever, cough, and sore throat’ [16]. The triad has sensitivity and specificity of, respectively, 67% and 64% against influenza confirmed by reverse transcriptase-polymerase chain reaction. For identifying possible COVID-19, we used a definition by Fulvio et al.: ‘concomitant presence of three or more of the following symptoms: fever, myalgia, cough, and smell or taste dysfunction’ [17]. This constellation of symptoms is highly likely to be associated with a positive molecular diagnosis of COVID-19 (odds ratio [OR] = 18.55; 95% confidence interval [CI]: 13.77–24.97) and has a specificity of 91.2%. Since the study was conducted after the pilgrims’ home return, collecting samples for laboratory testing could not be attempted.

The recorded data were analysed using Statistical Package for Social Sciences (SPSS) (IBM SPSS Statistics for Windows, version 26.0, IBM Corp, Armonk, NY, USA). For the descriptive analysis, the median with range was applied to summarise the continuous data, whereas frequencies and proportions were used to present the categorical variables as well as the prevalence of RTIs and possible ILI and possible COVID-19. The correlation of potential factors with the estimated prevalence of RTIs was carried out using simple logistic regression. The potential factors were chosen by reviewing the literature on respiratory infections and COVID-19 in Hajj and community settings [18,19]. The risk estimation was analysed using OR, and 95% CI and a *p*-value less than 0.05 was considered statistically significant.

## 3. Results

A total of 600 pilgrims were invited to participate, of whom 476 (79.3%) agreed to participate and completed the survey. The median age of the pilgrims was 50 (range 18–70) years (Table 1). About one quarter (25.4%) of pilgrims reported having one or more pre-existing medical conditions, and 422 (88.7%) received both doses of COVID-19 vaccine before commencing the Hajj journey (Table 2).

Eleven pilgrims (2.3%) reported developing one or more RTI symptoms two weeks after Hajj; of those, nine (81.8%) had rhinitis, six (54.5%) had a cough, five (45.5%) had a sore throat and two (18.2%) had dyspnoea with smell or taste dysfunction. Table 3 shows the breakdown of the individual and combinations of symptoms reported by pilgrims with RTIs. Of the symptomatic pilgrims, four (36.4%) sought treatment from a hospital or health clinic, three (27.3%) had come in contact with room-/tent-mates who developed RTIs during Hajj and five (45.5%) seem to have transmitted the infection to their household/workplace contacts after coming back from Hajj. The median duration of the symptoms was four (range 1–21) days, and their onset dates extended from the 23rd of July to the 1st of August 2021, i.e., from the last day to nine days after the consummation of Hajj rituals.

Only one participant met the definition of possible ILI, which translates to an estimated prevalence of 0.2%, and two met the definition of possible COVID-19, translating to an estimated prevalence of 0.4%; both of them were fully vaccinated and had no known history of COVID-19. In a simple logistic regression analysis, as illustrated in Table 4, there was no significant association between the prevalence of RTIs among pilgrims and the potential factors including age, gender, pre-existing medical conditions, education, smoking status, and receipts of influenza and COVID-19 vaccines (all *p* > 0.05).

## 4. Discussion

This study estimated the post-Hajj prevalence of symptomatic RTIs among pilgrims who attended the 2021 Hajj in the midst of the ongoing COVID-19 pandemic. The current study identified a low prevalence of possible ILI and possible COVID-19 among pilgrims. Rhinitis, cough and sore throat were the commonest clinical respiratory symptoms presented in our survey, and this was similar to what previous studies documented among Hajj pilgrims [4,5,20]. However, a cohort study during the same Hajj year showed greater prevalence rates of syndromic RTIs: 4.7% of the cohorts experienced RTI symptoms, and, respectively, 1.1% and 0.9% were suspected of having ILI and COVID-19. These lower rates were observed in this survey despite fewer pilgrims having university-level or higher education than those in the previous study (58.2% vs. 76.5%), and fewer were vaccinated against influenza (48.7% vs. 58.2%) and COVID-19 (88.7% vs. 92.5%) [15]. In contrast, previous studies found that the level of education was associated with higher compliance with preventive measures [21,22,23]. This discrepancy may have stemmed from recall bias in the current study, where the follow-up with pilgrims took place two weeks after Hajj, in contrast to the cohort study that was conducted in real-time during Hajj.

Almost half of the symptomatic pilgrims in this survey reported that one or more of their close contacts had developed similar RTI symptoms, which represents a high risk of contact transmission following Hajj. Previous studies that employed molecular diagnosis also showed the role of contact in the propagation of respiratory viral infection among Hajj pilgrims. For instance, a cross-sectional study involving UK pilgrims conducted in 2005 showed that 58% of pilgrims had a history of contact with ill pilgrims, and the frequency of influenza diagnosis was 17% among those who reported contact with URTI compared with 9% who reported no contact [24]. In a comparative study conducted the next year (in 2006), 7% of domestic Saudi pilgrims reported contact with an individual suffering from ILI, one of whom had laboratory-proven influenza, while 19% of UK pilgrims reported contact with individuals with ILI, of whom three had laboratory-proven influenza and two others had rhinovirus infection [25].

A significant proportion of pilgrims carry respiratory viruses and remain symptomatic upon their arrival to their home countries from Hajj, as was evidenced in several laboratory-based surveillance studies. In 2013, a study conducted among returning Hajj pilgrims at Kotoka International Airport in Ghana concluded that of all participants, 651 (77.6%) had respiratory symptoms, and 179 (21.3%) tested positive for one or more respiratory viruses [26]. In another surveillance conducted over two years (2014 and 2015) among returning Indian Hajj and Umrah pilgrims at Srinagar International Airport, Jammu and Kashmir, 300 pilgrims suffered from RTI, and 97 (32.3%) nasopharyngeal swabs were positive for a virus, most commonly influenza [27]. After Hajj 2006, a total of 255 pilgrims from Iran at Shiraz Airport experienced symptoms of RTI, with cough (83.5%) and sore throat (82%) being the commonest symptoms, and 83 (32.5%) had laboratory-proven viral infections [28].

The overall prevalence of RTIs among pilgrims in this Hajj season was markedly lower in comparison with the data reported in the pre-pandemic studies. For instance, a longitudinal survey conducted among domestic pilgrims from Riyadh city (capital of KSA) in 2010 showed that 53% developed RTIs [14]; similarly, ILI was recorded in 30% of Egyptian pilgrims in a survey from 2012 to 2015 [29], in 47.3% of French pilgrims in 2013 [30] and in 10% of Australian pilgrims in 2015 [18]. The previous studies were conducted prior to the pandemic, when up to 3.5 million pilgrims gathered to perform Hajj in inevitably overcrowded condition, with minimal physical distancing, and this may have in turn accelerated the transmission of RTIs [14,18,29,30]. Furthermore, the attendees of the recent Hajj seasons during the global pandemic may have become more aware of the important role of the non-pharmaceutical control measures against infections as a result of the intensive awareness-raising COVID-19 campaigns, although the pilgrims’ knowledge and practices of these interventions were not measured in this current survey.

The extent of COVID-19 related to the Hajj setting presented in our study was much lower than the KSA national data. A nationwide seroprevalence cross-sectional study estimated an overall prevalence of COVID-19 of 11% in KSA [31]. The stringent measures applied during Hajj including the disallowance of pilgrims aged ≥65 years may have contributed to the observed difference. In contrast, other down-scaled MGs (e.g., Tokyo Olympics 2020, Beijing Winter Olympics 2022) conducted using strict closed-loop and bubble systems in addition to COVID-19 vaccinations resulted only a sizeable outbreak but with no local transmission (Beijing Olympics), while during the Tokyo Olympics there was a high rate of local transmission [32,33]. Therefore, the success stories of the Hajj 2021 and the Beijing Winter Olympics 2022 imply that MGs can still be conducted amidst pandemics if carefully planned and managed with the implementation of stringent public health measures. This year (2022), a larger number of international and domestic pilgrims, roughly one million, performed Hajj safely without recording any outbreak of infectious diseases of global significance including COVID-19 and monkeypox [34].

The strength of this study is that to the best of our knowledge it is the only study that surveyed the health of pilgrims after Hajj amidst the COVID-19 pandemic. This study also has some limitations. It was conducted during an exceptional Hajj season when no overseas pilgrims were allowed unless they were already resident in KSA and pilgrims had to be healthy with no pre-existing diseases; therefore, the generalisability of the results is limited. Since we used the CATI method to collect data from participants, we ensured that we would not have missing data. However, some of the non-sensitive variables such as smoking status have a few missing data which are treated by available case analysis. Given that the study was conducted during COVID-19, the primary focus of this manuscript was on viral infection, but bacterial infection is also a possibility. A study conducted over 4 years (2014–2017) involving 485 French pilgrims showed that 33% of pilgrims acquired at least 1 respiratory virus, and 35% acquired at least 1 respiratory bacterium, suggesting that bacterial infections too are important [4]. Another limitation is that we used a standard definition of COVID-19 which may not capture atypical presentations of novel COVID-19 variants. Additionally, because this was a self-reported study, the participants’ responses may have been subjected to information bias. It is difficult to eliminate recall bias fully, but it could be minimised by a more careful selection of research questionnaires, giving intensive training to data collectors and allowing adequate time for participants to recall long-term memory. With the aim of further reducing recall bias, the duration of contact with roommates was not assessed. Since one fourth of SARS-CoV-2 infections remain asymptomatic [35], syndromic surveillance will miss a significant proportion of infections and thus will have limited value in controlling spread unless supplemented by a sensitive and specific diagnostic test. Finally, this was a syndromic survey, and the findings were not verified by microbiological tests. Large multinational follow-up studies are recommended for clinic-based syndromic surveillance, in conjunction with laboratory-based surveillance.

## 5. Conclusions

The results of this survey indicate that although the 2021 Hajj was conducted during COVID-19 challenges, the prevalence of symptomatic RTIs among the returning Hajj pilgrims was low, and only a small fraction of pilgrims met the syndromic definitions of ILI or COVID-19. Therefore, the COVID-19-related public health countermeasures during the Hajj seem to be a success story for conducting future MGs amidst pandemics, and adopting such changes can play an essential role in mitigating the transmission of COVID-19 and other RTIs as well as ensuring the safety and wellness of participants.

## Figures and Tables

**Table 1:** Participants’ socio-demographic characteristics (n = 476).

Characteristics	n (%)
Age (years)	
18–25	35 (7.4)
26–39	118 (24.8)
40–55	203 (42.7)
56–64	111 (23.4)
≥65	8 (1.7)
Gender	
Male:Female	257:219 (54:46)
Nationality	
Saudi Arabia	337 (70.8)
Egypt	29 (6.1)
Pakistan	27 (5.7)
India	21 (4.4)
Algeria	10 (2.1)
Others	52 (10.9)
Education	
No education	32 (6.7)
Primary/elementary school certificate	51 (10.7)
High school certificate	83 (17.4)
Diploma	33 (6.9)
Bachelor’s degree	206 (43.3)
Higher university degree	71 (14.9)

**Table 2:** Participants’ medical history and vaccination status (n = 476).

Variable	n (%)
Presence of pre-existing comorbidities	
Diabetes	67 (14.1)
Hypertension	62 (13)
Chronic lung diseases	11 (2.3)
Chronic heart diseases	6 (1.3)
Obesity	4 (0.8)
Others	6 (1.3)
Smoking status	
Non-smoker	359 (81.2)
Current smoker	46 (10.4)
Past smoker	29 (6.6)
Passive smoker	8 (1.8)
Influenza vaccine	
No	244 (51.3)
Yes	232 (48.7)
COVID-19 vaccine, dose 1	
No	7 (1.5)
Yes	469 (98.5)
COVID-19 vaccine, dose 2	
No	54 (11.3)
Yes	422 (88.7)

**Table 3:** Reported symptoms of respiratory tract infections among participants (n = 11).

**Individual Symptoms**	**n (%)**
Rhinitis	9 (81.8)
Fatigue	9 (81.8)
Cough	6 (54.5)
Sore throat	5 (45.5)
Subjective fever	4 (36.4)
Headache	4 (36.4)
Smell/taste dysfunction	2 (18.2)
Short of breath (dyspnoea)	2 (18.2)
Myalgia	2 (18.2)
Vomiting	1 (9.1)
**Combination of Symptoms**	**n (%)**
Subjective fever, and cough	1 (9.1)
Subjective fever, cough, sore throat, rhinitis, dyspnoea, smell/taste dysfunction, myalgia, fatigue, headache, and vomiting	1 (9.1)
Subjective fever, cough, dyspnoea, smell/taste dysfunction, myalgia, and fatigue	1 (9.1)
Subjective fever, cough, rhinitis, and fatigue	1 (9.1)
Cough, rhinitis, fatigue, and headache	2 (18.2)
Sore throat, rhinitis, and fatigue	2 (18.2)
Sore throat, rhinitis, and headache	1 (9.1)
Sore throat, rhinitis, fatigue, and headache	1 (9.1)
Rhinitis and fatigue	1 (9.1)

**Table 4:** Associations of potential factors with respiratory tract infections among participants (n = 11).

Variable	n (%)	OR (95% CI)	*p*-Value
Age (years)			
18–25	2 (5.7)	Reference	0.3
26–39	5 (4.2)	0.7 (0.01–3.9)	
40–55	2 (1)	0.2 (0.02–1.2)	
56–64	2 (1.8)	0.3 (0.04–2.2)	
≥65	0 (0)	0.0 (0.0–0.0)	
Gender			
Male	9 (1.9)	Reference	0.3
Female	6 (2.7)	1.4 (0.4–4.7)	
Education			
No education	1 (3.1)	1.1 (0.1–9)	0.4
Primary/elementary school certificate	0 (0)	0.0 (0.0–0.0)	
High school certificate	1 (1.2)	0.4 (0.1–3.3)	
Diploma	1 (3)	1.0 (0.1–8.7)	
University degree or higher	8 (2.9)	Reference	
Pre-existing medical conditions			
No	7 (2)	Reference	0.4
Yes	4 (3.3)	1.7 (0.5–5.9)	
Smoking status			
Non-smoker	9 (2.3)	Reference	0.4
Current smoker	2 (4.3)	1.9 (0.4–9.3)	
Influenza vaccine			
No	3 (1.2)	Reference	0.1
Yes	8 (3.4)	2.8 (0.7–10.9)	
COVID-19 vaccine			
No	1 (1.9)	Reference	0.8
Yes	10 (2.4)	1.3 (0.2–10.2)	

## Data Availability

The datasets generated and/or analysed during the current study are not publicly available due to containing information that could compromise participants’ privacy but are available from the corresponding author (HAM) on reasonable request.
